# Activities of Some Medicinal Plants on the Proliferation and Invasion of Brain Tumor Cell Lines

**DOI:** 10.1155/2020/3626879

**Published:** 2020-08-27

**Authors:** Saidu I. Ngulde, Umar K. Sandabe, Roger Abounader, Ying Zhang, Isa M. Hussaini

**Affiliations:** ^1^Department of Veterinary Pharmacology and Toxicology, Faculty of Veterinary, University of Maiduguri, PMB 1069, Maiduguri, Borno, Nigeria; ^2^Department of Veterinary Physiology and Biochemistry, Faculty of Veterinary, University of Maiduguri, PMB 1069, Maiduguri, Borno, Nigeria; ^3^Departments of Microbiology, Immunology and Cancer Biology, University of Virginia, PO Box 800168, Charlottesville, VA 22908, USA; ^4^Department of Pharmacology and Toxicology, Faculty of Pharmacy, University of Maiduguri, PMB 1069, Maiduguri, Borno, Nigeria

## Abstract

Cancer is a debilitating disease that is on the increase in both developed and developing countries. Anticancer drugs are often expensive, have narrow spectrum of activities, and are associated with toxicities and side effects such as myelosuppression, immunosuppression, gastrointestinal disturbance, alopecia, skin toxicity, and hepatotoxicity. Plants have been the major source of anticancer drugs both in orthodox and traditional medicine. Many of the plants claimed by the traditional medicine practitioners (TMPs) to be effective in the treatment of cancer are yet to be evaluated scientifically. In this work, five medicinal plants used by TMPs in Borno State, Nigeria, were tested against two brain tumor cell lines. Ethanol extracts of *Securidaca longepedunculata*, *Andira inermis* subsp. *rooseveltii*, *Annona senegalensis*, *Carissa edulis*, and *Parinari polyandra* were used. U87 and U231 brain tumor cell lines were used for proliferation assay, U251 cell line was used for the invasion assay in collagen V coated inserts, and U87 cell line was used for the western blot detection of cleaved Poly-ADP-Ribose-Polymerase (PARP). The result revealed that all tested extracts significantly (*p* < 0.05) inhibited the proliferation of U87 and U231 cell lines with the respective IC_50_ values ranging between 8 and 20 *μ*g/ml for *S. longepedunculata* and 100 and 90 *μ*g/ml for *P. polyandra*. The five extracts significantly (*p* < 0.05) inhibited the invasion of U251 cell line at the concentration of 10 *μ*g/ml (*S. longepedunculata*), 20 *μ*g/ml (*A. inermis*), 50 *μ*g/ml (*A. senegalensis*), 50 *μ*g/ml (*C. edulis*), and 50 *μ*g/ml (*P. polyandra*). *Securidaca longepedunculata* extract induced the cleavage of PARP. It was concluded that these medicinal plants have antiproliferative and anti-invasive activities and possess good prospects as source of anticancer agents especially *S. longepedunculata* which induced apoptosis in U87 cell line.

## 1. Introduction

Cancer is a debilitating disease that is on the increase in both developed and developing countries. It is the second leading cause of mortality after cardiovascular diseases in the U.S.A. In Nigeria, there are about 100,000 new cancer cases annually, and by the year 2020, the estimate would be 90.7 and 100.9 from every 100,000 Nigerian men and women, respectively [1–3]. Anticancer drugs are often expensive, have narrow spectrum of activities, and are associated with toxicities and side effects such as myelosuppression, immunosuppression, gastrointestinal disturbance, alopecia, skin toxicity, and hepatotoxicity.

Plants have been the major source of anticancer drugs both in orthodox and traditional medicine. The World Health Organization (WHO) estimated that approximately 60% of the world's inhabitants (and 80% of Africa's population) depends on herbal medicine for their primary health care [4]. The plants are the sources of antineoplastic drugs, vinblastine, and vincristine isolated from *Catharanthus roseus*, paclitaxel from *Taxus brevifol*, and camptothecin from *Camptotheca acuminata* which also lead to production of semisynthetic compounds, topotecan and irinotecan [5, 6].

Many of the plants claimed by the traditional medicine practitioners (TMPs) to be effective in the treatment of cancer are yet to be evaluated scientifically while some are undergoing scientific evaluations. The most commonly used plant in the Nigerian folklore medicine for treatment of cancer is *Securidaca longepedunculata* [7, 8]. It is used in the treatment of many other disease conditions and is regarded as the mother of all medicine. It is used in the control of insect pest. It has analgesic, antipyretic, anti-inflammatory, anticancer, antimicrobial, and hypoglycaemic properties. It is used in the treatment of malaria, toothache, rheumatism, epilepsy, constipation, and infertility in African traditional medicine. It is also used as fish poison and in the treatment of snake bites [8–11]. Many active compounds have been isolated from it which include xanthones (muchimangins), benzyl benzoates, methyl-salicylate, triterpene saponins, and bisdesmosidic saponins. Xanthones from *S. longepedunculata* have been reported for their cytotoxic and antitumor activities [12–16].

In this work, five medicinal plants including *S. longepedunculata* used by TMPs in Borno State were tested for their activities on proliferation and invasion in two brain tumor cell lines. Their activities on the cleavage of PARP were also assessed.

## 2. Materials and Methods

### 2.1. Collection of Plant Samples and Identification

The 5 plants were collected in Ngulde district in southern part of Borno State, Nigeria. They had been identified during a previous study [8]. Samples of plants collected include root barks of *Securidaca longepedunculata*, *Annona senegalensi*s, and *Carissa edulis* and stem barks of *Andira inermis* subsp. *rooseveltii* and *Parinari polyandra* during the dry season.

### 2.2. Preparation of Plant Extracts

Plant samples collected were air-dried at room temperature (25°C). Each sample was weighed several times until a constant weight obtained. The samples were pulverized in a mortar and pestle. About 1 kg of the ground herb was soaked overnight in petroleum ether. The residue from defatted samples was further extracted in 95% ethanol for 24 h. Each sample was filtered using Whatman filter paper No. 1 and desiccated to dryness under reduced pressure using a rotary evaporator.

### 2.3. Cell Lines and Cell Culture Conditions

Brain tumor cell lines (U87 and U251) were obtained from Dr Abounader's laboratory, University of Virginia Health System, Chalotesville, U.S.A. The U87 cancer cells were cultured in minimal essential medium-*α* (MEM-*α*) supplemented with 10% foetal bovine serum (FBS), 1% sodium pyruvate, 2% sodium bicarbonate, 1% nonessential amino acid, and 1% penicillin/streptomycin while the U251 cell line was cultured in (Roswell Park Memorial Institute) RPMI-1640 media supplemented with 5% FBS and 1% penicillin/streptomycin. The culture condition for all cells was 37°C and 5% CO_2_ in the presence of penicillin and streptomycin. Cells at 60–90% confluence were passaged.

### 2.4. Proliferation Assay

U87 and U231 cell lines were seeded in 6-well plates at 40,000 cells in triplicates using the standard media as described by Guessous et al. [17]. Cells were incubated overnight before treated with the extract at various concentrations depending on the extract but mostly at 3, 10, 30, 50, and 100 *μ*g/ml and then control wells. After 48 h of incubation, the cells were trypsinized, harvested, and counted using haemocytometer.

### 2.5. Invasion Assay

The U251 cell line was seeded in 10 mm^3^ Petri dishes at 200,000 cells per dish in 10 ml media. After 24 h incubation, cells were treated with the extract by replacing the media with the one containing the extract of *S. longepedunculata* (10 *μ*g/ml), *A. inermis* (20 *μ*g/ml), *A. senegalensis* (50 *μ*g/ml), *C. edulis* (50 *μ*g/ml), and *P. polyandra* (50 *μ*g/ml). This was incubated for 24 h including control (DMSO 0.05% v/v) dishes. Collagen V was kept at room temperature to be warmed and diluted with equal volume of sterile PBS to reduce the stock concentration to 250 *μ*g/ml. For each chamber, 300 *μ*l of collagen was dispensed and kept at room temperature in sterile condition overnight. This was to allow collagen to be coated on the chamber. Treated cells and controls were then harvested and seeded in collagen V coated inserts at 300,000 cells in 600 *μ*l of 0.1% FBS media (serum-free media). The normal (serum) media (600 *μ*l, 5% FBS) was aliquot into the lower chamber as chemoattractant as described by Guessous et al. [17]. After 8 h of incubation at 37°C, 5% CO_2_, the coated inserts were gently removed and their content discarded and rinsed with tap water and avoid touching the outer bottom. In a 12-well plate, 0.5 ml of 0.5% crystal violet was placed, and the inserts were immersed in the stain for 5 minutes. These were allowed to dry at room temperature overnight, viewed under the computer-aided N-180M biological microscope, and photomicrograph taken at x40 objectives. Cells within 5 different fields of equal dimensions per treatment were counted.

### 2.6. Western Blotting

The U87 cell line was seeded in 10 mm^3^ Petri dishes at 200,000 cells per dish in 10 ml media. After 24 h incubation, cells were treated with the extract by replacing the media with the one containing the extracts: *S. longepedunculata* (10 *μ*g/ml), *A. inermis* (20 *μ*g/ml), and *A. senegalensis* (50 *μ*g/ml). It was incubated for 24 and 48 h including control dishes. Cells were harvested, and protein contents were extracted using the RIPA buffer (1% Igepal, 0.5% sodium deoxycholate, 0.1% SDS in PBS). Extracted proteins were kept at −20°C for further use. Protein concentration was determined using Comassie plus reagent (Bradford assay), and absorbance was taken using the ELISA reader.

The western blot was performed as described by Zhang et al. [18]. Equal amounts of proteins were loaded and separated in a sodium dodecyl sulfate-polyacrylamide gel electrophoresis (SDS-PAGE) (Invitrogen, Carlsbad, CA) at 140 V (initial setting at 60 V for 20 minutes) for 1.5 h at room temperature and then transferred to a nitrocellulose membrane at 35 V for 1.75 h at 4°C. The membrane was rinsed in TBST and then blocked with 5% skimmed milk in TBST for 1 h at room temperature. The membrane was incubated with the primary antibodies (anti-PARP, anti-cleaved PARP, and anti-actin) in fresh 5% skimmed milk TBST at 4°C overnight on a slow shaker. The antibody-bound membranes were washed 3 times in TBST each for 10 minutes. They were then treated with the specific secondary antibody in 5% skimmed milk TBST and incubated for 1 h at room temperature on a slow shaker followed by washing 3 times. The immunoreactive signals were detected with super signal ultrachemiluminescent substrate (Thermo Scientific, Rockford, IL, USA).

### 2.7. Statistics

Cells were exposed to extracts in triplicates, and results obtained were expressed as mean ± standard error of the mean (SEM) and analysed by one-way analysis of variance (ANOVA). Microsoft Excel (2011) for Mac and Graphpad Prism® version 4.00 for Windows were used for data presentation and analyses. *p* < 0.05 was considered significant.

## 3. Results

### 3.1. Screening for Antiproliferative Activities of Five Plant Extracts on U87 Cell Line

**Figure 1:**
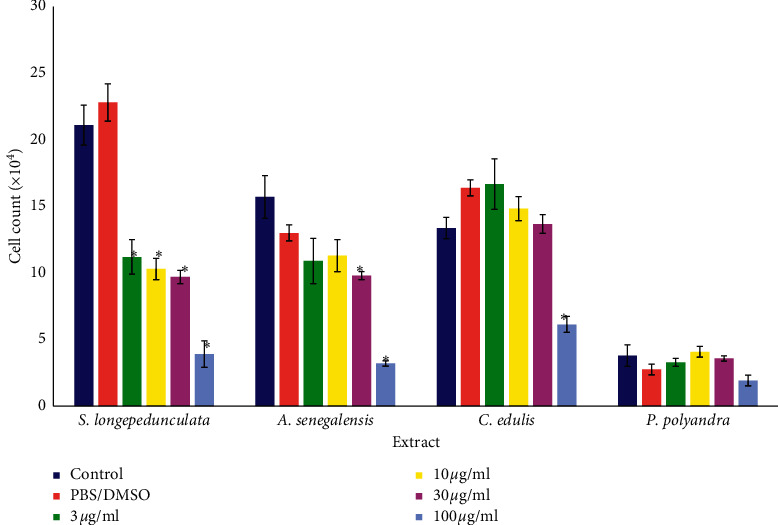
Effect of various plant extracts on the proliferation of U87. ^*∗*^*p* < 0.05 compared with the control or PBS group. There is concentration-dependent inhibition by administration of *S. longepedunculata* extract 48 h after treatment on U87 cell lines. Significant (*p* < 0.05) antiproliferation is observed from 3 *μ*g/ml on U87 and 10 *μ*g/ml in U251 cell lines. PBS was used in *S. longepedunculata*, and DMSO (≤1:1000) was used in all other extracts. U87 cells were exposed to *A. inermis* at 25, 50, and 100 *μ*g/ml with significant inhibition at 50 *μ*g/ml. The concentrations are different from the one in the figure and could not be presented together.

**Figure 2:**
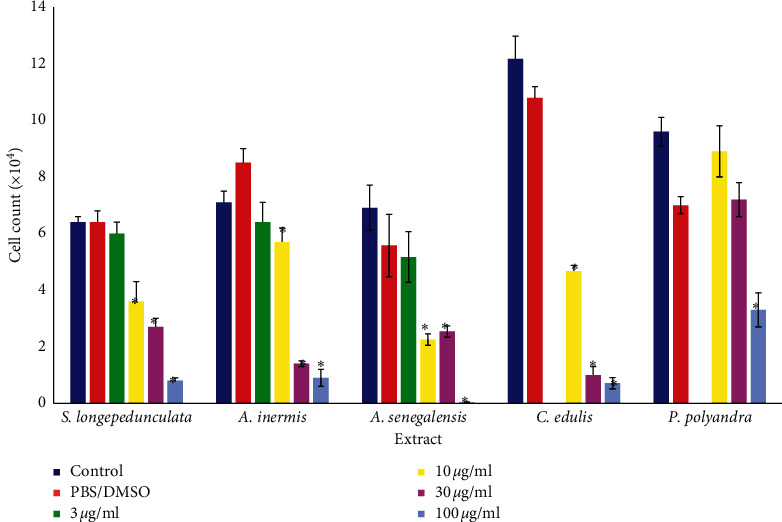
Effect of various plant extracts on the proliferation of U251. ^*∗*^*p* < 0.05 compared with the control or PBS group. There is concentration-dependent inhibition by administration of the extracts 48 h after treatment on U251 cell lines. Significant (*p* < 0.05) antiproliferation is observed from 10 *μ*g/ml in by all extracts except *P. polyandra* which is at 100 *μ*g/ml. PBS was used in *S. longepedunculata*, and DMSO (≤1:1000) was used in all other extracts.

The result of antiproliferation of 5 plant extracts on U87 cell line is presented in Figure 1. Concentration-dependent activities of *S. longepedunculata* extract 48 h after treatment with significant (*p* < 0.05) antiproliferation were observed from 3 *μ*g/ml (11.2 × 10^4^ ± 1.3) when compared with the negative control well (21.1 × 10^4^ ± 1.5). The other extracts inhibited proliferation with significant activities from 30 *μ*g/ml for *A. senegalensis* and 100 *μ*g/ml for *C. edulis* while *P. polyandra* produced nonsignificant inhibition. The activities of the extracts on the proliferation of U251 as presented in Figure 2 indicated that all five extracts were significantly (*p* < 0.05) active from 10 *μ*g/ml with the exception of *P. polyandra* that showed significant activities at 100 *μ*g/ml only as compared with that of the respective control wells. *Securidaca longepedunculata*, *A. senegalensis*, and *C. edulis* recorded concentration-dependent antiproliferative activities on U251 cell line. The respective IC_50_ values were determined and presented in Table 1 as 8 and 20 *μ*g/ml for *S. longepedunculata* on U87 and U251, 20 and 20 *μ*g/ml for *A. inermis* on U87 and U251, 60 and 10 *μ*g/ml for *A. senegalensis* on U87 and U251, 90 and 8 *μ*g/ml for *C. edulis* on U87 and U251, and 100 and 90 *μ*g/ml for *P. polyandra* on U87 and U251.

### 3.2. Effects of Various Extracts on the Invasion of U251 Cell Line

**Figure 3:**
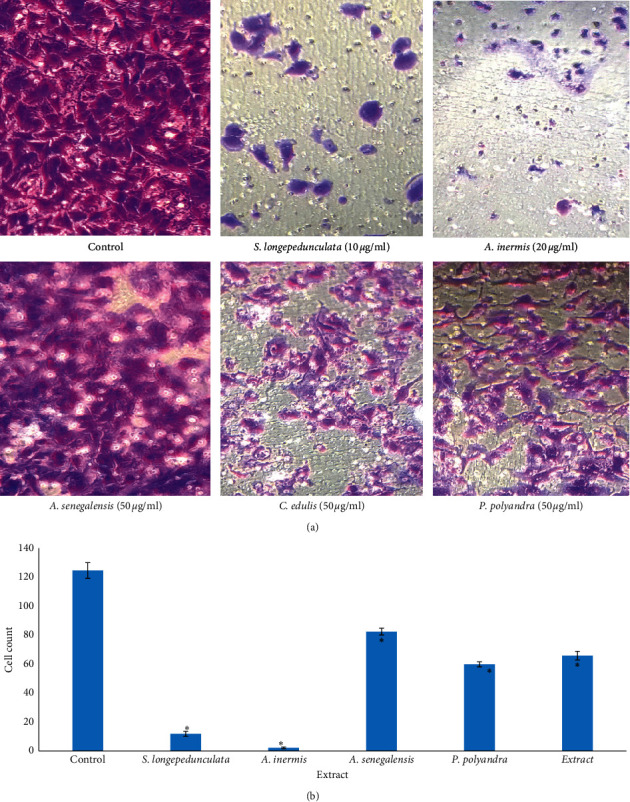
Effect of various plant extracts on the invasion of U251 cell line. ^*∗*^*p* < 0.05 compared with the control. All extracts inhibited invasion significantly (*p* < 0.05). Highest activities are in *A. inermis* and *S. longepedunculata*.

Figure 3 presents the effect of various plant extracts on the invasion through the collagen V-coated membrane. All extracts significantly (*p* < 0.05) inhibited the invasion of U251 cell line. Highest inhibition of invasion was observed in wells treated with *A. inermis* and *S. longepedunculata* which were significantly (*p* < 0.05) greater than other extract-treated wells and control.

### 3.3. Effect of Extracts on Apoptosis

**Figure 4:**
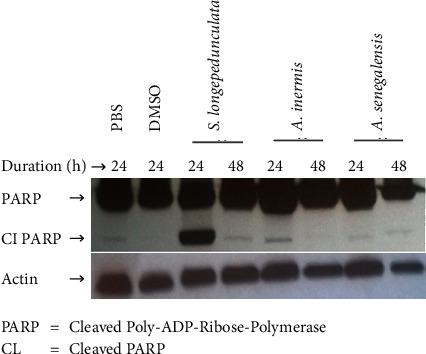
Effect of ethanol extracts *S. longepedunculata*, *A. inermis*, and *A. senegalensis* on induction of PARP protein in U87 cell line. PARP was cleaved in cells treated with *S. longepedunculata* root bark extract only.

Figure 4 shows the western blot analysis of U87 cell line after treating with various extracts. There was expression of cleaved PARP (Poly-ADP-Ribose-Polymerase) in cells treated with extract of *S. longepedunculata* while no expression was there in the cells treated with *A. inermis* and *A. senegalensis.*

## 4. Discussion

In this work, five medicinal plants were screened for their antiproliferative activities and possible anticancer activities. These plant extracts were selected based on the previous work, which indicated that they were used by traditional medicine practitioners (TMPs) in Borno State, Nigeria, for management of cancers [8]. All five extracts produced significant (*p* < 0.05) antiproliferative effects on one or both of the U87 and U251 cell lines used. In U87 cell line, the genetic defect affects PTEN while in U251 cell line, the genetic defects affect both PTEN and TP53. Highest activities against U87 were recorded in the decreasing order as follows *S. longependunculata*, *A. inermis*, *A. senegalensis, C. edulis*, and *P. polyandra*. In contrast, the order of activity against U251 changed to *C. edulis*, *A. senegalensis*, *S. longepedunculata*, *A. inermis*, and *P. polyandra*. Both PTEN and TP53 are tumor suppressor genes. The difference in the degree of antiproliferation on the two cell lines may suggest that the different extracts act through different mechanisms. *Carissa spinarum* (*C. edulis*) has been reported to induce caspase 3/7 activity and cell cycle arrest in melanoma cells [19]. In brain tumor cell line U1242, *C. edulis* caused antiproliferation via epidermal growth factor (EGF) with IC_50_ of 1.74 *μ*g/ml [20]. This is better than the results presented here on the U87 and U251 cell lines.

*Securidaca longepedunculata* produced concentration-dependent significant activities from 3 *μ*g/ml on U87 and 10 *μ*g/ml on U251 cell lines 48 h after treatment. When these extracts were tested for their effects on invasion using collagen V-coated membrane, all extracts significantly (*p* < 0.05) inhibited the invasion of U251 cell line within 8 h of invasion with *A. inermis* and *S. longepedunculata* producing greatest effects. The effects of the extracts on proliferation and invasion may be why the plants are useful to TMPs in the management of cancers. The previous study conducted showed that *S. longepedunculata* root aqueous extract has cytotoxic effects on Ehrlich ascites carcinoma with IC_50_ of 67 *μ*g/ml [21] which is much higher than the results from this study with IC_50_ of 8 and 20 *μ*g/ml on U87 and U251, respectively.

When the extracts were tested on the induction of cleaved Poly-ADP-Ribose-Polymerase (PARP), only *S. longepedunculata* extract had activity indicating there could be induction of apoptosis by this extract. This further supports the potential of *S. longepedunculata* for its use in the treatment of cancer. This is in agreement with the work of Ngulde et al. [22] where *S. longepedunculata* induced cleavage of PARP in U87 cell line. Also, Obasi et al. [15] reported that saponins from *Securidaca longepedunculata* were found to induce apoptosis and inhibit migration and invasion in cervical cancer cells. Xanthones isolated from the plant inhibited the proliferation of lung cancer cell line and acted as an inducer of apoptosis [14].

## 5. Conclusion

In conclusion, this study showed the five plant extracts had activities against brain tumor cell lines during proliferation and invasion. *Securidaca longepedunculata* had the highest activities in inhibiting proliferation and invasion and is the only one which induced cleavage of PARP. There is the need for further research including in vivo study to verify the potentials and prospect of these plant extracts for the folkloric usage in treating cancers.

## Figures and Tables

**Table 1:** Median inhibitory concentrations (IC _50_) of various extracts on U87 and U251 cell lines.

Extract	U87 (*μ*g/ml)	U251 (*μ*g/ml)
*S. longepedunculata*	8	20
*A. inermis*	20	20
*A. senegalensis*	60	10
*C. edulis*	90	8
*P. polyandra*	100	90

## Data Availability

The data are available from the corresponding author upon request.

## References

[1] Parkin D. M., Ferlay J., Cherif M. (2003). *Cancer in Africa Epidemiology and Prevention*.

[2] World Health Organization (2008). The impact of cancer-Nigeria. http://www.who.int/infobase/report.aspxAccessedon17/03/12.

[3] Omolara K. A. (2011). Feasible cancer control strategies for Nigeria: mini-review. *American Journal of Tropical Medicine and Public Health*.

[4] World Health Organization (2000). *General Guidelines for Methodologies on Research and Evaluation of Traditional Medicine*.

[5] Kingston D. G. I., Cragg G. M., Kingston D. G. I., Newman D. J. (2012). Taxols and its analogs. *Anticancer Agents from Natural Products*.

[6] Cragg G. M., Newman D. J. (2005). Plants as a source of anti-cancer agents. *Journal of Ethnopharmacology*.

[7] Ngulde S. I., Sandabe U. K., Hussaini I. M. (2015). Ethnobotanical survey of anticancer plants in Askira/Uba local government area of Borno State, Nigeria. *African Journal of Pharmacy and Pharmacology*.

[8] Segun P. A., Ogbole O. O., Ajaiyeoba E. O. (2018). Medicinal plants used in the management of cancer among the Ijebus of Southwestern Nigeria. *Journal of Herbal Medicine*.

[9] Stevenson P. C., Dayarathna T. K., Belmain S. R., Veitch N. C. (2009). Bisdesmosidic saponins from *Securidaca longepedunculata* roots: evaluation of deterrency and toxicity to coleopteran storage pests. *Journal of Agricultural and Food Chemistry*.

[10] Ngulde S. I., Fomnya H. J., Tijjani M. B. (2013). Acute Toxicity and antinociceptive activity of crude ethanol extract of *Securidaca longepedunculata* (Fresen) root bark in albino rats. *International Journal of Drug Research and Technology*.

[11] Keshebo D. L., Choudhury M. K., Hussen A., Bekele T. Antimicrobial activities of *Securidaca longipedunculata* (polygalaceae) and isolation of benzyl 2-hydroxy-5-methoxy benzoate.

[12] Mitaine-Offer A. C., Pénez N., Miyamoto T. (2010). Acylated triterpene saponins from the roots of *Securidaca longepedunculata*. *Phytochemistry*.

[13] Dibwe D. F., Awale S., Kadota S., Morita H., Tezuka Y., Muchimangin G. J. (2014). Muchimangins G. J, fully substituted xanthones with a diphenylmethyl substituent, from *Securidaca longepedunculata*. *Journal of Natural Products*.

[14] Jian Zuo J., Jiang H., Zhu Y. H., Wang Y. Q., Zhang W., Luan J. J. (2016). Regulation of MAPKs signaling contributes to the growth inhibition of 1,7-Dihydroxy-3,4-dimethoxyxanthone on multidrug resistance A549/taxol cells. *Evidence-Based Complementary and Alternative Medicine*.

[15] Obasi T., Braicu C., Iacob B. C. (2018). Securidaca–saponins are natural inhibitors of AKT, MCL-1, and BCL2L1 in cervical cancer cells. *Cancer Management and Research*.

[16] Klein-Júnior L. C., Campos A., Niero R., Corrêa R., Heyden Y. V. (2019). Xanthones and cancer: From natural sources to mechanisms of action. *Chemistry and Biodiversity*.

[17] Guessous F., Alvarado M., Marcinkiewicz L. (2013). Oncogenic effects of miR-10b in glioblastoma stem cells. *Journal of Neurooncology*.

[18] Zhang Y., Zhang L., Wang F. (2011). Activation of M3 muscarinic receptors inhibits T-type Ca^2+^ channel currents via pertussis toxin-sensitive novel protein kinase C pathway in small dorsal root ganglion neurons. *Cell Signaling*.

[19] Alqathama A., Bader A., Khondkar P., Gibbons S., Prieto J. (2015). Bioguided isoloation of cytotoxic compounds against melanoma cells from *Carissa spinarum* L. *Planta Medica*.

[20] Ya’u J., Magaji M. G., Yaro A. H. (2016). Antitumour properties of the standardised root bark extract of *Carissa edulis* Vahl. *Nigerian Journal of Pharmaceutical Sciences*.

[21] Lawal R. A., Ozaslan M. D., Odesanmi O. S., Karagoz I. D., Kilic I. H., Ebuehi O. A. T. (2012). Cytotoxic and antiproliferative activity of *Securidaca longepedunculata* aqueous extract on *Ehrlich ascites* carcinoma cells in swiss albino mice. *International Journal of Applied Research in Natural Products*.

[22] Ngulde S. I., Sandabe U. K., Abounader R. (2019). Ethanol extract of *Securidaca longipedunculata* induces apoptosis in brain tumor (U87) cells. *BioMed Research International*.

